# Supplementary material for: A scalable online tool for quantitative social network assessment reveals potentially modifiable social environmental risks
Source: Nat Commun. 2018 Sep 26;9:3930. doi: 10.1038/s41467-018-06408-6 (PMC6158181; doi:10.1038/s41467-018-06408-6)
Supplement: Supplementary file 1 — Supplementary Information [file 41467_2018_6408_MOESM1_ESM.pdf]

A Scalable Online Tool for Quantitative Social Network Assessment Reveals Potentially Modifiable Social Environmental Risks

Dhand et al.

Supplementary Methods 1: Social Network Questionnaire

GEMS Social Network Survey

Welcome to the GEMS Social Network Survey!

This survey is about your social network and its potential health effects.

Please carefully follow the instructions, particularly in the begining of this survey as it impacts all future questions.

We estimate that the survey will take about 10-20 minutes.

To ensure confidentiality, individual names, nicknames, and initials listed by the participant will not be used in publications and will not be shared publicly.

Please tell us a little bit about yourself first.

Date of birth ______________________________
(YYYY-MM-DD)

Age ______________________________
(Please do not proceed with this survey if you are younger than 18 years old. This study can only enroll participants 18 years and older.)

Gender
◯ Female
◯ Male

What is your current address? ______________________________
(e.g. 123 Main Street Boston MA)

What is your zip code? ______________________________

What is your employment status?	◯ Employed for wages<br>◯ Self-employed<br>◯ Out of work and looking for work<br>◯ Out of work but not currently looking for work<br>◯ A homemaker<br>◯ A student<br>◯ Military<br>◯ Retired<br>◯ Unable to work<br>◯ Prefer not to answer
Education level	◯ 1-8 years<br>◯ Some high school<br>◯ High school grad<br>◯ Some college<br>◯ Associate degree<br>◯ Bachelor's degree<br>◯ Graduate degree<br>◯ Prefer not to answer
Marital status	◯ Not married<br>◯ Married
Do you live alone?	◯ Yes<br>◯ No
Have you been diagnosed with Multiple Sclerosis?	◯ Yes<br>◯ No
How old were you when you first noticed neurological symptoms related to Multiple Sclerosis?	________________________________<br>(Symptoms may be difficulty with seeing, speaking, using arms or legs, walking, numbness or tingling. Please only refer to symptoms that lasted at least 24 hours.)
How old were you when you were diagnosed?	________________________________

Your Functional Status

Below is a scale that attempts to rate the severity of MS symptoms, please rate your current functioning:

	0 No symptoms: "I have no symptoms or disability in this specific area"	1 Some symptoms, no disability: "I am aware of symptoms but no limits on my activities"	2 Mild disability: "I have mild limits on my activities, but I do not need help from others or to use other aides"	3 Moderate disability: "I have moderate limits on my activities and I sometimes need help from others or use other aides"	4 Severe disability: "I have severe limits on my activities and I usually need help from others or use other aides"
Walking	○	○	○	○	○
Using your arms and hands	○	○	○	○	○
Vision	○	○	○	○	○
Speech	○	○	○	○	○
Swallowing	○	○	○	○	○
Thinking, memory or cognition	○	○	○	○	○
Numbness, tingling, burning sensation or pain	○	○	○	○	○
Controlling your bladder and/or bowel	○	○	○	○	○

We are now going to ask you to name people in your life. Please only use first names, nicknames, or initials of adults (older than 18). If two people have the same first name, then add a last initial. Please list up to 5 names in the boxes, and more names in the big box after. Don't worry about duplicates OR blanks. We will deal with them later.

From time to time, most people discuss important personal matters with other people. Looking back over the last 3 months, who are the adults with whom you discussed an important personal matter? Please list the names of these adults.

Name 1 ______________________________

Name 2 ______________________________

Name 3 ______________________________

Name 4 ______________________________

Name 5 ______________________________

More names, separated by commas (if needed):

____

From time to time, people socialize with other people. For instance, they visit each other, go together on a trip or to a dinner. In the last 3 months, who are the adults with whom you usually do these things? Please list their first names. Don't worry about duplicates OR blanks.

Name 1 ______________________

Name 2 ______________________

Name 3 ______________________

Name 4 ______________________

Name 5 ______________________

More names, separated by commas (if needed):

____

Think about people who support you to stay healthy. In the last 3 months, who provides support for your health needs? Please list their first names. Don't worry about duplicates OR blanks.

Name 1 ______________________

Name 2 ______________________

Name 3 ______________________

Name 4 ______________________

Name 5 ______________________

More names, separated by commas (if needed):

____

Now, we are going to create a unique list of names by removing all duplicate names and blanks.

Please review the full list of names you provided. Only the first 5 names for each section are shown.

If any person appears more than once or there is a blank line, click the REMOVE button next to the name to remove duplicate names or blanks. Otherwise click the KEEP button. Each person should appear only once on the list below.

You must provide a choice ("Keep" or "Remove") for every line before moving on. When you have made a choice for every line, then move to the next question.

	Remove	Keep
[name1]	○	○
[name2]	○	○
[name3]	○	○
[name4]	○	○
[name5]	○	○
[name6]	○	○
[name7]	○	○
[name8]	○	○
[name9]	○	○
[name10]	○	○
[name11]	○	○
[name12]	○	○
[name13]	○	○
[name14]	○	○
[name15]	○	○

Number Filled in First 10 Slots ________________________________
(Number you kept in first 10 slots)

Total number of social contacts ________________________________
(Total number of names you kept.)

We are now going to focus on the relationships in your social network. If you notice some names missing, that is okay.

Below should be a unique list of names without blanks. If that is not the case, please stop and go back.

We are interested in how close you feel to each person below. Compared to the others you mentioned, how close do you feel to each person?

[name1]	◯ especially close<br>◯ not especially close
[name2]	◯ especially close<br>◯ not especially close
[name3]	◯ especially close<br>◯ not especially close
[name4]	◯ especially close<br>◯ not especially close
[name5]	◯ especially close<br>◯ not especially close
[name6]	◯ especially close<br>◯ not especially close
[name7]	◯ especially close<br>◯ not especially close
[name8]	◯ especially close<br>◯ not especially close
[name9]	◯ especially close<br>◯ not especially close
[name10]	◯ especially close<br>◯ not especially close
[name11]	◯ especially close<br>◯ not especially close
[name12]	◯ especially close<br>◯ not especially close
[name13]	◯ especially close<br>◯ not especially close
[name14]	◯ especially close<br>◯ not especially close
[name15]	◯ especially close<br>◯ not especially close

We will now focus on the relationship between each pair of people you mentioned. For example, we will ask about [name1] and [name2]. For each pair, we will ask whether they are total strangers, in-between, or especially close. Here is what we mean by each term:

Total strangers: The two people wouldn't recognize one another if they met on the street.

In-between: Relationships in the middle of total strangers and especially close. Typically, these people are casual acquaintances.

Especially close: As close or closer to each other than they are to you.

Take your time. This can be difficult.

Is [name1] a total stranger, especially close, or in-between with [name2]?	◯ stranger<br>◯ in-between<br>◯ especially close
Is [name1] a total stranger, especially close, or in-between with [name3]?	◯ stranger<br>◯ in-between<br>◯ especially close
Is [name1] a total stranger, especially close, or in-between with [name4]?	◯ stranger<br>◯ in-between<br>◯ especially close
Is [name1] a total stranger, especially close, or in-between with [name5]?	◯ stranger<br>◯ in-between<br>◯ especially close
Is [name1] a total stranger, especially close, or in-between with [name6]?	◯ stranger<br>◯ in-between<br>◯ especially close
Is [name1] a total stranger, especially close, or in-between with [name7]?	◯ stranger<br>◯ in-between<br>◯ especially close
Is [name1] a total stranger, especially close, or in-between with [name8]?	◯ stranger<br>◯ in-between<br>◯ especially close
Is [name1] a total stranger, especially close, or in-between with [name9]?	◯ stranger<br>◯ in-between<br>◯ especially close
Is [name1] a total stranger, especially close, or in-between with [name10]?	◯ stranger<br>◯ in-between<br>◯ especially close
Is [name1] a total stranger, especially close, or in-between with [name11]?	◯ stranger<br>◯ in-between<br>◯ especially close
Is [name1] a total stranger, especially close, or in-between with [name12]?	◯ stranger<br>◯ in-between<br>◯ especially close
Is [name1] a total stranger, especially close, or in-between with [name13]?	◯ stranger<br>◯ in-between<br>◯ especially close
Is [name1] a total stranger, especially close, or in-between with [name14]?	◯ stranger<br>◯ in-between<br>◯ especially close
Is [name1] a total stranger, especially close, or in-between with [name15]?	◯ stranger<br>◯ in-between<br>◯ especially close
Is [name2] a total stranger, especially close, or in-between with [name3]?	◯ stranger<br>◯ in-between<br>◯ especially close
Is [name2] a total stranger, especially close, or in-between with [name4]?	◯ stranger<br>◯ in-between<br>◯ especially close

Is [name2] a total stranger, especially close, or in-between with [name5]?
- ◯ stranger
- ◯ in-between
- ◯ especially close

Is [name2] a total stranger, especially close, or in-between with [name6]?
- ◯ stranger
- ◯ in-between
- ◯ especially close

Is [name2] a total stranger, especially close, or in-between with [name7]?
- ◯ stranger
- ◯ in-between
- ◯ especially close

Is [name2] a total stranger, especially close, or in-between with [name8]?
- ◯ stranger
- ◯ in-between
- ◯ especially close

Is [name2] a total stranger, especially close, or in-between with [name9]?
- ◯ stranger
- ◯ in-between
- ◯ especially close

Is [name2] a total stranger, especially close, or in-between with [name10]?
- ◯ stranger
- ◯ in-between
- ◯ especially close

Is [name2] a total stranger, especially close, or in-between with [name11]?
- ◯ stranger
- ◯ in-between
- ◯ especially close

Is [name2] a total stranger, especially close, or in-between with [name12]?
- ◯ stranger
- ◯ in-between
- ◯ especially close

Is [name2] a total stranger, especially close, or in-between with [name13]?
- ◯ stranger
- ◯ in-between
- ◯ especially close

Is [name2] a total stranger, especially close, or in-between with [name14]?
- ◯ stranger
- ◯ in-between
- ◯ especially close

Is [name2] a total stranger, especially close, or in-between with [name15]?
- ◯ stranger
- ◯ in-between
- ◯ especially close

Is [name3] a total stranger, especially close, or in-between with [name4]?
- ◯ stranger
- ◯ in-between
- ◯ especially close

Is [name3] a total stranger, especially close, or in-between with [name5]?
- ◯ stranger
- ◯ in-between
- ◯ especially close

Is [name3] a total stranger, especially close, or in-between with [name6]?
- ◯ stranger
- ◯ in-between
- ◯ especially close

Is [name3] a total stranger, especially close, or in-between with [name7]?
- ◯ stranger
- ◯ in-between
- ◯ especially close

Is [name3] a total stranger, especially close, or in-between with [name8]?
- ◯ stranger
- ◯ in-between
- ◯ especially close

Is [name3] a total stranger, especially close, or in-between with [name9]?
- ◯ stranger
- ◯ in-between
- ◯ especially close

Is [name3] a total stranger, especially close, or in-between with [name10]?
- ◯ stranger
- ◯ in-between
- ◯ especially close

Is [name3] a total stranger, especially close, or in-between with [name11]?
- ◯ stranger
- ◯ in-between
- ◯ especially close

Is [name3] a total stranger, especially close, or in-between with [name12]?
- ◯ stranger
- ◯ in-between
- ◯ especially close

Is [name3] a total stranger, especially close, or in-between with [name13]?
- ◯ stranger
- ◯ in-between
- ◯ especially close

Is [name3] a total stranger, especially close, or in-between with [name14]?
- ◯ stranger
- ◯ in-between
- ◯ especially close

Is [name3] a total stranger, especially close, or in-between with [name15]?
- ◯ stranger
- ◯ in-between
- ◯ especially close

Is [name4] a total stranger, especially close, or in-between with [name5]?
- ◯ stranger
- ◯ in-between
- ◯ especially close

Is [name4] a total stranger, especially close, or in-between with [name6]?
- ◯ stranger
- ◯ in-between
- ◯ especially close

Is [name4] a total stranger, especially close, or in-between with [name7]?
- ◯ stranger
- ◯ in-between
- ◯ especially close

Is [name4] a total stranger, especially close, or in-between with [name8]?
- ◯ stranger
- ◯ in-between
- ◯ especially close

Is [name4] a total stranger, especially close, or in-between with [name9]?
- ◯ stranger
- ◯ in-between
- ◯ especially close

Is [name4] a total stranger, especially close, or in-between with [name10]?
- ◯ stranger
- ◯ in-between
- ◯ especially close

Is [name4] a total stranger, especially close, or in-between with [name11]?
- ◯ stranger
- ◯ in-between
- ◯ especially close

Is [name4] a total stranger, especially close, or in-between with [name12]?
- ◯ stranger
- ◯ in-between
- ◯ especially close

Is [name4] a total stranger, especially close, or in-between with [name13]?
- ◯ stranger
- ◯ in-between
- ◯ especially close

Is [name4] a total stranger, especially close, or in-between with [name14]?
- ◯ stranger
- ◯ in-between
- ◯ especially close

Is [name4] a total stranger, especially close, or in-between with [name15]?
- ◯ stranger
- ◯ in-between
- ◯ especially close

Is [name5] a total stranger, especially close, or in-between with [name6]?
- ◯ stranger
- ◯ in-between
- ◯ especially close

Is [name5] a total stranger, especially close, or in-between with [name7]?
- ◯ stranger
- ◯ in-between
- ◯ especially close

Is [name5] a total stranger, especially close, or in-between with [name8]?
- ◯ stranger
- ◯ in-between
- ◯ especially close

Is [name5] a total stranger, especially close, or in-between with [name9]?
- ◯ stranger
- ◯ in-between
- ◯ especially close

Is [name5] a total stranger, especially close, or in-between with [name10]?
- ◯ stranger
- ◯ in-between
- ◯ especially close

Is [name5] a total stranger, especially close, or in-between with [name11]?
- ◯ stranger
- ◯ in-between
- ◯ especially close

Is [name5] a total stranger, especially close, or in-between with [name12]?
- ◯ stranger
- ◯ in-between
- ◯ especially close

Is [name5] a total stranger, especially close, or in-between with [name13]?
- ◯ stranger
- ◯ in-between
- ◯ especially close

Is [name5] a total stranger, especially close, or in-between with [name14]?
- ◯ stranger
- ◯ in-between
- ◯ especially close

Is [name5] a total stranger, especially close, or in-between with [name15]?
- ◯ stranger
- ◯ in-between
- ◯ especially close

Is [name6] a total stranger, especially close, or in-between with [name7]?
- ◯ stranger
- ◯ in-between
- ◯ especially close

Is [name6] a total stranger, especially close, or in-between with [name8]?
- ◯ stranger
- ◯ in-between
- ◯ especially close

Is [name6] a total stranger, especially close, or in-between with [name9]?
- ◯ stranger
- ◯ in-between
- ◯ especially close

Is [name6] a total stranger, especially close, or in-between with [name10]?
- ◯ stranger
- ◯ in-between
- ◯ especially close

Is [name6] a total stranger, especially close, or in-between with [name11]?
- ◯ stranger
- ◯ in-between
- ◯ especially close

Is [name6] a total stranger, especially close, or in-between with [name12]?
- ◯ stranger
- ◯ in-between
- ◯ especially close

Is [name6] a total stranger, especially close, or in-between with [name13]?
- ◯ stranger
- ◯ in-between
- ◯ especially close

Is [name6] a total stranger, especially close, or in-between with [name14]?
- ◯ stranger
- ◯ in-between
- ◯ especially close

Is [name6] a total stranger, especially close, or in-between with [name15]?
- ◯ stranger
- ◯ in-between
- ◯ especially close

Is [name7] a total stranger, especially close, or in-between with [name8]?
- ◯ stranger
- ◯ in-between
- ◯ especially close

Is [name7] a total stranger, especially close, or in-between with [name9]?
- ◯ stranger
- ◯ in-between
- ◯ especially close

Is [name7] a total stranger, especially close, or in-between with [name10]?
- ◯ stranger
- ◯ in-between
- ◯ especially close

Is [name7] a total stranger, especially close, or in-between with [name11]?
- ◯ stranger
- ◯ in-between
- ◯ especially close

Is [name7] a total stranger, especially close, or in-between with [name12]?
- ◯ stranger
- ◯ in-between
- ◯ especially close

Is [name7] a total stranger, especially close, or in-between with [name13]?
- ◯ stranger
- ◯ in-between
- ◯ especially close

Is [name7] a total stranger, especially close, or in-between with [name14]?
- ◯ stranger
- ◯ in-between
- ◯ especially close

Is [name7] a total stranger, especially close, or in-between with [name15]?
- ◯ stranger
- ◯ in-between
- ◯ especially close

Is [name8] a total stranger, especially close, or in-between with [name9]?
- ◯ stranger
- ◯ in-between
- ◯ especially close

Is [name8] a total stranger, especially close, or in-between with [name10]?
- ◯ stranger
- ◯ in-between
- ◯ especially close

Is [name8] a total stranger, especially close, or in-between with [name11]?
- ◯ stranger
- ◯ in-between
- ◯ especially close

Is [name8] a total stranger, especially close, or in-between with [name12]?
- ◯ stranger
- ◯ in-between
- ◯ especially close

Is [name8] a total stranger, especially close, or in-between with [name13]?
- ◯ stranger
- ◯ in-between
- ◯ especially close

Is [name8] a total stranger, especially close, or in-between with [name14]?
- ◯ stranger
- ◯ in-between
- ◯ especially close

Is [name8] a total stranger, especially close, or in-between with [name15]?
- ◯ stranger
- ◯ in-between
- ◯ especially close

Is [name9] a total stranger, especially close, or in-between with [name10]?
- ◯ stranger
- ◯ in-between
- ◯ especially close

Is [name9] a total stranger, especially close, or in-between with [name11]?
- ◯ stranger
- ◯ in-between
- ◯ especially close

Is [name9] a total stranger, especially close, or in-between with [name12]?
- ◯ stranger
- ◯ in-between
- ◯ especially close

Is [name9] a total stranger, especially close, or in-between with [name13]?
- ◯ stranger
- ◯ in-between
- ◯ especially close

Is [name9] a total stranger, especially close, or in-between with [name14]?
- ◯ stranger
- ◯ in-between
- ◯ especially close

Is [name9] a total stranger, especially close, or in-between with [name15]?
- ◯ stranger
- ◯ in-between
- ◯ especially close

Is [name10] a total stranger, especially close, or in-between with [name11]?
- ◯ stranger
- ◯ in-between
- ◯ especially close

Is [name10] a total stranger, especially close, or in-between with [name12]?
- ◯ stranger
- ◯ in-between
- ◯ especially close

Is [name10] a total stranger, especially close, or in-between with [name13]?
- ◯ stranger
- ◯ in-between
- ◯ especially close

Is [name10] a total stranger, especially close, or in-between with [name14]?
- ◯ stranger
- ◯ in-between
- ◯ especially close

Is [name10] a total stranger, especially close, or in-between with [name15]?
- ◯ stranger
- ◯ in-between
- ◯ especially close

Is [name11] a total stranger, especially close, or in-between with [name12]?
- ◯ stranger
- ◯ in-between
- ◯ especially close

Is [name11] a total stranger, especially close, or in-between with [name13]?

◯ stranger
◯ in-between
◯ especially close

Is [name11] a total stranger, especially close, or in-between with [name14]?

◯ stranger
◯ in-between
◯ especially close

Is [name11] a total stranger, especially close, or in-between with [name15]?

◯ stranger
◯ in-between
◯ especially close

Is [name12] a total stranger, especially close, or in-between with [name13]?

◯ stranger
◯ in-between
◯ especially close

Is [name12] a total stranger, especially close, or in-between with [name14]?

◯ stranger
◯ in-between
◯ especially close

Is [name12] a total stranger, especially close, or in-between with [name15]?

◯ stranger
◯ in-between
◯ especially close

Is [name13] a total stranger, especially close, or in-between with [name14]?

◯ stranger
◯ in-between
◯ especially close

Is [name13] a total stranger, especially close, or in-between with [name15]?

◯ stranger
◯ in-between
◯ especially close

Is [name14] a total stranger, especially close, or in-between with [name15]?

◯ stranger
◯ in-between
◯ especially close

We're now going to ask about how much support you have from people in general. If you needed it, how often is someone available to do the following for you?

To help you if you were confined to bed?

◯ None of the time
◯ A little of the time
◯ Some of the time
◯ Most of the time
◯ All of the time

To take you to the doctor if you need it?

◯ None of the time
◯ A little of the time
◯ Some of the time
◯ Most of the time
◯ All of the time

To prepare your meals if you are unable to do it yourself?

◯ None of the time
◯ A little of the time
◯ Some of the time
◯ Most of the time
◯ All of the time

To help with daily chores if you were sick?

- ◯ None of the time
- ◯ A little of the time
- ◯ Some of the time
- ◯ Most of the time
- ◯ All of the time

To have a good time with?

- ◯ None of the time
- ◯ A little of the time
- ◯ Some of the time
- ◯ Most of the time
- ◯ All of the time

To turn for suggestions about how to deal with a personal problem?

- ◯ None of the time
- ◯ A little of the time
- ◯ Some of the time
- ◯ Most of the time
- ◯ All of the time

Who understands your problems?

- ◯ None of the time
- ◯ A little of the time
- ◯ Some of the time
- ◯ Most of the time
- ◯ All of the time

To love and make you feel wanted?

- ◯ None of the time
- ◯ A little of the time
- ◯ Some of the time
- ◯ Most of the time
- ◯ All of the time

Out of the people listed below, which person or persons do these supportive actions most often? You can chose more than one person.

	Supports me most often
[name1]	☐
[name2]	☐
[name3]	☐
[name4]	☐
[name5]	☐
[name6]	☐
[name7]	☐
[name8]	☐
[name9]	☐
[name10]	☐
[name11]	☐
[name12]	☐
[name13]	☐
[name14]	☐
[name15]	☐

What gender is each person in your social network?

	Male	Female	Other
[name1]	○	○	○
[name2]	○	○	○
[name3]	○	○	○
[name4]	○	○	○
[name5]	○	○	○
[name6]	○	○	○
[name7]	○	○	○
[name8]	○	○	○
[name9]	○	○	○
[name10]	○	○	○
[name11]	○	○	○
[name12]	○	○	○
[name13]	○	○	○
[name14]	○	○	○
[name15]	○	○	○

Do any people have a negative influence on your health? For example, does a person passively or actively encourage you to smoke, not eat well, or not exercise?

[name1] ○ Yes ○ No

[name2] ○ Yes ○ No

[name3] ○ Yes ○ No

[name4] ○ Yes ○ No

[name5] ○ Yes ○ No

[name6] ○ Yes ○ No

[name7] ○ Yes ○ No

[name8] ○ Yes ○ No

[name9] ○ Yes ○ No

[name10] ○ Yes ○ No

[name11]	◯ Yes<br>◯ No
[name12]	◯ Yes<br>◯ No
[name13]	◯ Yes<br>◯ No
[name14]	◯ Yes<br>◯ No
[name15]	◯ Yes<br>◯ No

What is the race of each person in your social network?

	White	Black	Asian	American Indian	Native Hawaiian	Other	Don't know
[name1]	◯	◯	◯	◯	◯	◯	◯
[name2]	◯	◯	◯	◯	◯	◯	◯
[name3]	◯	◯	◯	◯	◯	◯	◯
[name4]	◯	◯	◯	◯	◯	◯	◯
[name5]	◯	◯	◯	◯	◯	◯	◯
[name6]	◯	◯	◯	◯	◯	◯	◯
[name7]	◯	◯	◯	◯	◯	◯	◯
[name8]	◯	◯	◯	◯	◯	◯	◯
[name9]	◯	◯	◯	◯	◯	◯	◯
[name10]	◯	◯	◯	◯	◯	◯	◯
[name11]	◯	◯	◯	◯	◯	◯	◯
[name12]	◯	◯	◯	◯	◯	◯	◯
[name13]	◯	◯	◯	◯	◯	◯	◯
[name14]	◯	◯	◯	◯	◯	◯	◯
[name15]	◯	◯	◯	◯	◯	◯	◯

What is the ethnicity of each person in your social network?

	Not Hispanic or Latino	Hispanic or Latino	Don't know
[name1]	○	○	○
[name2]	○	○	○
[name3]	○	○	○
[name4]	○	○	○
[name5]	○	○	○
[name6]	○	○	○
[name7]	○	○	○
[name8]	○	○	○
[name9]	○	○	○
[name10]	○	○	○
[name11]	○	○	○
[name12]	○	○	○
[name13]	○	○	○
[name14]	○	○	○
[name15]	○	○	○

As far as you know, what is the highest level of education of each person?

	1-8 years	Some high school	High school grad	Some college	Associate degree	Bachelors degree	Graduate degree	Don't know
[name1]	○	○	○	○	○	○	○	○
[name2]	○	○	○	○	○	○	○	○
[name3]	○	○	○	○	○	○	○	○
[name4]	○	○	○	○	○	○	○	○
[name5]	○	○	○	○	○	○	○	○
[name6]	○	○	○	○	○	○	○	○
[name7]	○	○	○	○	○	○	○	○
[name8]	○	○	○	○	○	○	○	○
[name9]	○	○	○	○	○	○	○	○
[name10]	○	○	○	○	○	○	○	○
[name11]	○	○	○	○	○	○	○	○
[name12]	○	○	○	○	○	○	○	○
[name13]	○	○	○	○	○	○	○	○
[name14]	○	○	○	○	○	○	○	○
[name15]	○	○	○	○	○	○	○	○

On average, how often do you speak with each person in your network?

	Daily	Weekly	Monthly	Less often	Don't know
[name1]	○	○	○	○	○
[name2]	○	○	○	○	○
[name3]	○	○	○	○	○
[name4]	○	○	○	○	○
[name5]	○	○	○	○	○
[name6]	○	○	○	○	○
[name7]	○	○	○	○	○
[name8]	○	○	○	○	○
[name9]	○	○	○	○	○
[name10]	○	○	○	○	○
[name11]	○	○	○	○	○
[name12]	○	○	○	○	○
[name13]	○	○	○	○	○
[name14]	○	○	○	○	○
[name15]	○	○	○	○	○

How long have you known the following people (in years)?

	Less than three	Three to six	More than six	Don't know
[name1]	○	○	○	○
[name2]	○	○	○	○
[name3]	○	○	○	○
[name4]	○	○	○	○
[name5]	○	○	○	○
[name6]	○	○	○	○
[name7]	○	○	○	○
[name8]	○	○	○	○
[name9]	○	○	○	○
[name10]	○	○	○	○
[name11]	○	○	○	○
[name12]	○	○	○	○
[name13]	○	○	○	○
[name14]	○	○	○	○
[name15]	○	○	○	○

We're now going to ask all the ways each person is connected to you. Some people can be connected to you in more than one way. For example, a man could be your brother and he could belong to your church and be your lawyer. For each person below, please tell us all the ways that person is connected to you. Co-member in organization: Someone who is a member of the same organization as you. For example, a person who attends the same church**

	Spouse	Parent	Sibling	Child	Other family	Co-worker	Comem - ber in org**	Neigh-bor	Friend	Advisor
[name1]	☐	☐	☐	☐	☐	☐	☐	☐	☐	☐
[name2]	☐	☐	☐	☐	☐	☐	☐	☐	☐	☐
[name3]	☐	☐	☐	☐	☐	☐	☐	☐	☐	☐
[name4]	☐	☐	☐	☐	☐	☐	☐	☐	☐	☐
[name5]	☐	☐	☐	☐	☐	☐	☐	☐	☐	☐
[name6]	☐	☐	☐	☐	☐	☐	☐	☐	☐	☐
[name7]	☐	☐	☐	☐	☐	☐	☐	☐	☐	☐
[name8]	☐	☐	☐	☐	☐	☐	☐	☐	☐	☐
[name9]	☐	☐	☐	☐	☐	☐	☐	☐	☐	☐
[name10]	☐	☐	☐	☐	☐	☐	☐	☐	☐	☐
[name11]	☐	☐	☐	☐	☐	☐	☐	☐	☐	☐
[name12]	☐	☐	☐	☐	☐	☐	☐	☐	☐	☐
[name13]	☐	☐	☐	☐	☐	☐	☐	☐	☐	☐
[name14]	☐	☐	☐	☐	☐	☐	☐	☐	☐	☐
[name15]	☐	☐	☐	☐	☐	☐	☐	☐	☐	☐

Has anyone been diagnosed with MS?

	Yes	No	Don't know
[name1]	○	○	○
[name2]	○	○	○
[name3]	○	○	○
[name4]	○	○	○
[name5]	○	○	○
[name6]	○	○	○
[name7]	○	○	○
[name8]	○	○	○
[name9]	○	○	○
[name10]	○	○	○
[name11]	○	○	○

[name12]	◯	◯	◯
[name13]	◯	◯	◯
[name14]	◯	◯	◯
[name15]	◯	◯	◯

How many years ago was the diagnosis of MS?

If within this year, then round up to 1.

[name1] ____________________

[name2] ____________________

[name3] ____________________

[name4] ____________________

[name5] ____________________

[name6] ____________________

[name7] ____________________

[name8] ____________________

[name9] ____________________

[name10] ____________________

[name11] ____________________

[name12] ____________________

[name13] ____________________

[name14] ____________________

[name15] ____________________

Please select the best option that applies to [name1]'s mobility.

- ◯ Normal: functionally normal with no limitations on activity or lifestyle_ May have minor abnormality on examination _ Course is relapsing-remitting with a return to baseline with or without treatment_ Are not treated with any ongoing therapy for MS
- ◯ Mild disability: mild symptoms or signs_ Have definite findings such as sensory abnormalities, mild bladder impairment, minor incoordination, weakness, or fatigue_ No visible abnormality of walking_ The pattern of disease is relapsing-remitting, but may not have a full return to baseline following attacks_ May use ongoing therapy such as amantadine, baclofen, or oxybutynin
- ◯ Moderate disability: visibly abnormal walking but do not require walking aids _ The pattern of disease is relapsing-remitting or progressive
- ◯ Early cane: intermittent use of cane_ Other forms of unilateral support including splint, brace, or crutch _ Use unilateral support primarily for longer distances, but are able to walk at least 25 feet without it_ The pattern of disease is relapsing-remitting or progressive
- ◯ Late cane: dependent on a cane or other forms of unilateral support and cannot walk 25 feet without such support _ Patients may hang on to furniture inside their homes or touch the wall when walking in clinic_ May use a scooter for greater distances (e.g., malls) _ Pattern of disease is relapsing remitting or progressive
- ◯ Bilateral support: requires bilateral support to walk 25 feet_ May use two canes or two crutches or a walker _ May use a scooter for greater distances_ Pattern of disease is relapsing-remitting or progressive
- ◯ Confined to wheelchair: essentially confined to a wheelchair or scooter _ May be able to take a few steps but are unable to ambulate 25 feet, even with bilateral support _ May show further progression including worsening hand function or inability to transfer independently
- ◯ Unclassifiable: does not fit the above classifications_ Significant memory or visual impairment, overwhelming fatigue, or significant bowel or bladder impairment in an otherwise minimally impaired patient

Please select the best option that applies to [name2]'s mobility.

○ Normal: functionally normal with no limitations on activity or lifestyle_ May have minor abnormality on examination _ Course is relapsing-remitting with a return to baseline with or without treatment_ Are not treated with any ongoing therapy for MS
○ Mild disability: mild symptoms or signs_ Have definite findings such as sensory abnormalities, mild bladder impairment, minor incoordination, weakness, or fatigue_ No visible abnormality of walking_ The pattern of disease is relapsing-remitting, but may not have a full return to baseline following attacks_ May use ongoing therapy such as amantadine, baclofen, or oxybutynin
○ Moderate disability: visibly abnormal walking but do not require walking aids _ The pattern of disease is relapsing-remitting or progressive
○ Early cane: intermittent use of cane_ Other forms of unilateral support including splint, brace, or crutch _ Use unilateral support primarily for longer distances, but are able to walk at least 25 feet without it_ The pattern of disease is relapsing-remitting or progressive
○ Late cane: dependent on a cane or other forms of unilateral support and cannot walk 25 feet without such support _ Patients may hang on to furniture inside their homes or touch the wall when walking in clinic_ May use a scooter for greater distances (e.g., malls) _ Pattern of disease is relapsing remitting or progressive
○ Bilateral support: requires bilateral support to walk 25 feet_ May use two canes or two crutches or a walker _ May use a scooter for greater distances_ Pattern of disease is relapsing-remitting or progressive
○ Confined to wheelchair: essentially confined to a wheelchair or scooter _ May be able to take a few steps but are unable to ambulate 25 feet, even with bilateral support _ May show further progression including worsening hand function or inability to transfer independently
○ Unclassifiable: does not fit the above classifications_ Significant memory or visual impairment, overwhelming fatigue, or significant bowel or bladder impairment in an otherwise minimally impaired patient

Please select the best option that applies to [name3]'s mobility.

○ Normal: functionally normal with no limitations on activity or lifestyle_ May have minor abnormality on examination _ Course is relapsing-remitting with a return to baseline with or without treatment_ Are not treated with any ongoing therapy for MS
○ Mild disability: mild symptoms or signs_ Have definite findings such as sensory abnormalities, mild bladder impairment, minor incoordination, weakness, or fatigue_ No visible abnormality of walking_ The pattern of disease is relapsing-remitting, but may not have a full return to baseline following attacks_ May use ongoing therapy such as amantadine, baclofen, or oxybutynin
○ Moderate disability: visibly abnormal walking but do not require walking aids _ The pattern of disease is relapsing-remitting or progressive
○ Early cane: intermittent use of cane_ Other forms of unilateral support including splint, brace, or crutch _ Use unilateral support primarily for longer distances, but are able to walk at least 25 feet without it_ The pattern of disease is relapsing-remitting or progressive
○ Late cane: dependent on a cane or other forms of unilateral support and cannot walk 25 feet without such support _ Patients may hang on to furniture inside their homes or touch the wall when walking in clinic_ May use a scooter for greater distances (e.g., malls) _ Pattern of disease is relapsing remitting or progressive
○ Bilateral support: requires bilateral support to walk 25 feet_ May use two canes or two crutches or a walker _ May use a scooter for greater distances_ Pattern of disease is relapsing-remitting or progressive
○ Confined to wheelchair: essentially confined to a wheelchair or scooter _ May be able to take a few steps but are unable to ambulate 25 feet, even with bilateral support _ May show further progression including worsening hand function or inability to transfer independently
○ Unclassifiable: does not fit the above classifications_ Significant memory or visual impairment, overwhelming fatigue, or significant bowel or bladder impairment in an otherwise minimally impaired patient

Please select the best option that applies to [name4]'s mobility.

○ Normal: functionally normal with no limitations on activity or lifestyle_ May have minor abnormality on examination _ Course is relapsing-remitting with a return to baseline with or without treatment_ Are not treated with any ongoing therapy for MS
○ Mild disability: mild symptoms or signs_ Have definite findings such as sensory abnormalities, mild bladder impairment, minor incoordination, weakness, or fatigue_ No visible abnormality of walking_ The pattern of disease is relapsing-remitting, but may not have a full return to baseline following attacks_ May use ongoing therapy such as amantadine, baclofen, or oxybutynin
○ Moderate disability: visibly abnormal walking but do not require walking aids _ The pattern of disease is relapsing-remitting or progressive
○ Early cane: intermittent use of cane_ Other forms of unilateral support including splint, brace, or crutch _ Use unilateral support primarily for longer distances, but are able to walk at least 25 feet without it_ The pattern of disease is relapsing-remitting or progressive
○ Late cane: dependent on a cane or other forms of unilateral support and cannot walk 25 feet without such support _ Patients may hang on to furniture inside their homes or touch the wall when walking in clinic_ May use a scooter for greater distances (e.g., malls) _ Pattern of disease is relapsing remitting or progressive
○ Bilateral support: requires bilateral support to walk 25 feet_ May use two canes or two crutches or a walker _ May use a scooter for greater distances_ Pattern of disease is relapsing-remitting or progressive
○ Confined to wheelchair: essentially confined to a wheelchair or scooter _ May be able to take a few steps but

are unable to ambulate 25 feet, even with bilateral support _ May show further progression including worsening hand function or inability to transfer independently
◯ Unclassifiable: does not fit the above classifications_ Significant memory or visual impairment, overwhelming fatigue, or significant bowel or bladder impairment in an otherwise minimally impaired patient

Please select the best option that applies to [name5]'s mobility.

◯ Normal: functionally normal with no limitations on activity or lifestyle_ May have minor abnormality on examination _ Course is relapsing-remitting with a return to baseline with or without treatment_ Are not treated with any ongoing therapy for MS
◯ Mild disability: mild symptoms or signs_ Have definite findings such as sensory abnormalities, mild bladder impairment, minor incoordination, weakness, or fatigue_ No visible abnormality of walking_ The pattern of disease is relapsing-remitting, but may not have a full return to baseline following attacks_ May use ongoing therapy such as amantadine, baclofen, or oxybutynin
◯ Moderate disability: visibly abnormal walking but do not require walking aids _ The pattern of disease is relapsing-remitting or progressive
◯ Early cane: intermittent use of cane_ Other forms of unilateral support including splint, brace, or crutch _ Use unilateral support primarily for longer distances, but are able to walk at least 25 feet without it_ The pattern of disease is relapsing-remitting or progressive
◯ Late cane: dependent on a cane or other forms of unilateral support and cannot walk 25 feet without such support _ Patients may hang on to furniture inside their homes or touch the wall when walking in clinic_ May use a scooter for greater distances (e.g., malls) _ Pattern of disease is relapsing remitting or progressive
◯ Bilateral support: requires bilateral support to walk 25 feet_ May use two canes or two crutches or a walker _ May use a scooter for greater distances_ Pattern of disease is relapsing-remitting or progressive
◯ Confined to wheelchair: essentially confined to a wheelchair or scooter _ May be able to take a few steps but are unable to ambulate 25 feet, even with bilateral support _ May show further progression including worsening hand function or inability to transfer independently
◯ Unclassifiable: does not fit the above classifications_ Significant memory or visual impairment, overwhelming fatigue, or significant bowel or bladder impairment in an otherwise minimally impaired patient

Please select the best option that applies to [name6]'s mobility.

◯ Normal: functionally normal with no limitations on activity or lifestyle_ May have minor abnormality on examination _ Course is relapsing-remitting with a return to baseline with or without treatment_ Are not treated with any ongoing therapy for MS
◯ Mild disability: mild symptoms or signs_ Have definite findings such as sensory abnormalities, mild bladder impairment, minor incoordination, weakness, or fatigue_ No visible abnormality of walking_ The pattern of disease is relapsing-remitting, but may not have a full return to baseline following attacks_ May use ongoing therapy such as amantadine, baclofen, or oxybutynin
◯ Moderate disability: visibly abnormal walking but do not require walking aids _ The pattern of disease is relapsing-remitting or progressive
◯ Early cane: intermittent use of cane_ Other forms of unilateral support including splint, brace, or crutch _ Use unilateral support primarily for longer distances, but are able to walk at least 25 feet without it_ The pattern of disease is relapsing-remitting or progressive
◯ Late cane: dependent on a cane or other forms of unilateral support and cannot walk 25 feet without such support _ Patients may hang on to furniture inside their homes or touch the wall when walking in clinic_ May use a scooter for greater distances (e.g., malls) _ Pattern of disease is relapsing remitting or progressive
◯ Bilateral support: requires bilateral support to walk 25 feet_ May use two canes or two crutches or a walker _ May use a scooter for greater distances_ Pattern of disease is relapsing-remitting or progressive
◯ Confined to wheelchair: essentially confined to a wheelchair or scooter _ May be able to take a few steps but are unable to ambulate 25 feet, even with bilateral support _ May show further progression including worsening hand function or inability to transfer independently
◯ Unclassifiable: does not fit the above classifications_ Significant memory or visual impairment, overwhelming fatigue, or significant bowel or bladder impairment in an otherwise minimally impaired patient

Please select the best option that applies to [name7]'s mobility.

○ Normal: functionally normal with no limitations on activity or lifestyle_ May have minor abnormality on examination _ Course is relapsing-remitting with a return to baseline with or without treatment_ Are not treated with any ongoing therapy for MS
○ Mild disability: mild symptoms or signs_ Have definite findings such as sensory abnormalities, mild bladder impairment, minor incoordination, weakness, or fatigue_ No visible abnormality of walking_ The pattern of disease is relapsing-remitting, but may not have a full return to baseline following attacks_ May use ongoing therapy such as amantadine, baclofen, or oxybutynin
○ Moderate disability: visibly abnormal walking but do not require walking aids _ The pattern of disease is relapsing-remitting or progressive
○ Early cane: intermittent use of cane_ Other forms of unilateral support including splint, brace, or crutch _ Use unilateral support primarily for longer distances, but are able to walk at least 25 feet without it_ The pattern of disease is relapsing-remitting or progressive
○ Late cane: dependent on a cane or other forms of unilateral support and cannot walk 25 feet without such support _ Patients may hang on to furniture inside their homes or touch the wall when walking in clinic_ May use a scooter for greater distances (e.g., malls) _ Pattern of disease is relapsing remitting or progressive
○ Bilateral support: requires bilateral support to walk 25 feet_ May use two canes or two crutches or a walker _ May use a scooter for greater distances_ Pattern of disease is relapsing-remitting or progressive
○ Confined to wheelchair: essentially confined to a wheelchair or scooter _ May be able to take a few steps but are unable to ambulate 25 feet, even with bilateral support _ May show further progression including worsening hand function or inability to transfer independently
○ Unclassifiable: does not fit the above classifications_ Significant memory or visual impairment, overwhelming fatigue, or significant bowel or bladder impairment in an otherwise minimally impaired patient

Please select the best option that applies to [name8]'s mobility.

○ Normal: functionally normal with no limitations on activity or lifestyle_ May have minor abnormality on examination _ Course is relapsing-remitting with a return to baseline with or without treatment_ Are not treated with any ongoing therapy for MS
○ Mild disability: mild symptoms or signs_ Have definite findings such as sensory abnormalities, mild bladder impairment, minor incoordination, weakness, or fatigue_ No visible abnormality of walking_ The pattern of disease is relapsing-remitting, but may not have a full return to baseline following attacks_ May use ongoing therapy such as amantadine, baclofen, or oxybutynin
○ Moderate disability: visibly abnormal walking but do not require walking aids _ The pattern of disease is relapsing-remitting or progressive
○ Early cane: intermittent use of cane_ Other forms of unilateral support including splint, brace, or crutch _ Use unilateral support primarily for longer distances, but are able to walk at least 25 feet without it_ The pattern of disease is relapsing-remitting or progressive
○ Late cane: dependent on a cane or other forms of unilateral support and cannot walk 25 feet without such support _ Patients may hang on to furniture inside their homes or touch the wall when walking in clinic_ May use a scooter for greater distances (e.g., malls) _ Pattern of disease is relapsing remitting or progressive
○ Bilateral support: requires bilateral support to walk 25 feet_ May use two canes or two crutches or a walker _ May use a scooter for greater distances_ Pattern of disease is relapsing-remitting or progressive
○ Confined to wheelchair: essentially confined to a wheelchair or scooter _ May be able to take a few steps but are unable to ambulate 25 feet, even with bilateral support _ May show further progression including worsening hand function or inability to transfer independently
○ Unclassifiable: does not fit the above classifications_ Significant memory or visual impairment, overwhelming fatigue, or significant bowel or bladder impairment in an otherwise minimally impaired patient

Please select the best option that applies to [name9]'s mobility.

○ Normal: functionally normal with no limitations on activity or lifestyle_ May have minor abnormality on examination _ Course is relapsing-remitting with a return to baseline with or without treatment_ Are not treated with any ongoing therapy for MS
○ Mild disability: mild symptoms or signs_ Have definite findings such as sensory abnormalities, mild bladder impairment, minor incoordination, weakness, or fatigue_ No visible abnormality of walking_ The pattern of disease is relapsing-remitting, but may not have a full return to baseline following attacks_ May use ongoing therapy such as amantadine, baclofen, or oxybutynin
○ Moderate disability: visibly abnormal walking but do not require walking aids _ The pattern of disease is relapsing-remitting or progressive
○ Early cane: intermittent use of cane_ Other forms of unilateral support including splint, brace, or crutch _ Use unilateral support primarily for longer distances, but are able to walk at least 25 feet without it_ The pattern of disease is relapsing-remitting or progressive
○ Late cane: dependent on a cane or other forms of unilateral support and cannot walk 25 feet without such support _ Patients may hang on to furniture inside their homes or touch the wall when walking in clinic_ May use a scooter for greater distances (e.g., malls) _ Pattern of disease is relapsing remitting or progressive
○ Bilateral support: requires bilateral support to walk 25 feet_ May use two canes or two crutches or a walker _ May use a scooter for greater distances_ Pattern of disease is relapsing-remitting or progressive
○ Confined to wheelchair: essentially confined to a wheelchair or scooter _ May be able to take a few steps but

are unable to ambulate 25 feet, even with bilateral support _ May show further progression including worsening hand function or inability to transfer independently
◯ Unclassifiable: does not fit the above classifications_ Significant memory or visual impairment, overwhelming fatigue, or significant bowel or bladder impairment in an otherwise minimally impaired patient

Please select the best option that applies to [name10]'s mobility.

◯ Normal: functionally normal with no limitations on activity or lifestyle_ May have minor abnormality on examination _ Course is relapsing-remitting with a return to baseline with or without treatment_ Are not treated with any ongoing therapy for MS
◯ Mild disability: mild symptoms or signs_ Have definite findings such as sensory abnormalities, mild bladder impairment, minor incoordination, weakness, or fatigue_ No visible abnormality of walking_ The pattern of disease is relapsing-remitting, but may not have a full return to baseline following attacks_ May use ongoing therapy such as amantadine, baclofen, or oxybutynin
◯ Moderate disability: visibly abnormal walking but do not require walking aids _ The pattern of disease is relapsing-remitting or progressive
◯ Early cane: intermittent use of cane_ Other forms of unilateral support including splint, brace, or crutch _ Use unilateral support primarily for longer distances, but are able to walk at least 25 feet without it_ The pattern of disease is relapsing-remitting or progressive
◯ Late cane: dependent on a cane or other forms of unilateral support and cannot walk 25 feet without such support _ Patients may hang on to furniture inside their homes or touch the wall when walking in clinic_ May use a scooter for greater distances (e.g., malls) _ Pattern of disease is relapsing remitting or progressive
◯ Bilateral support: requires bilateral support to walk 25 feet_ May use two canes or two crutches or a walker _ May use a scooter for greater distances_ Pattern of disease is relapsing-remitting or progressive
◯ Confined to wheelchair: essentially confined to a wheelchair or scooter _ May be able to take a few steps but are unable to ambulate 25 feet, even with bilateral support _ May show further progression including worsening hand function or inability to transfer independently
◯ Unclassifiable: does not fit the above classifications_ Significant memory or visual impairment, overwhelming fatigue, or significant bowel or bladder impairment in an otherwise minimally impaired patient

Please select the best option that applies to [name11]'s mobility.

◯ Normal: functionally normal with no limitations on activity or lifestyle_ May have minor abnormality on examination _ Course is relapsing-remitting with a return to baseline with or without treatment_ Are not treated with any ongoing therapy for MS
◯ Mild disability: mild symptoms or signs_ Have definite findings such as sensory abnormalities, mild bladder impairment, minor incoordination, weakness, or fatigue_ No visible abnormality of walking_ The pattern of disease is relapsing-remitting, but may not have a full return to baseline following attacks_ May use ongoing therapy such as amantadine, baclofen, or oxybutynin
◯ Moderate disability: visibly abnormal walking but do not require walking aids _ The pattern of disease is relapsing-remitting or progressive
◯ Early cane: intermittent use of cane_ Other forms of unilateral support including splint, brace, or crutch _ Use unilateral support primarily for longer distances, but are able to walk at least 25 feet without it_ The pattern of disease is relapsing-remitting or progressive
◯ Late cane: dependent on a cane or other forms of unilateral support and cannot walk 25 feet without such support _ Patients may hang on to furniture inside their homes or touch the wall when walking in clinic_ May use a scooter for greater distances (e.g., malls) _ Pattern of disease is relapsing remitting or progressive
◯ Bilateral support: requires bilateral support to walk 25 feet_ May use two canes or two crutches or a walker _ May use a scooter for greater distances_ Pattern of disease is relapsing-remitting or progressive
◯ Confined to wheelchair: essentially confined to a wheelchair or scooter _ May be able to take a few steps but are unable to ambulate 25 feet, even with bilateral support _ May show further progression including worsening hand function or inability to transfer independently
◯ Unclassifiable: does not fit the above classifications_ Significant memory or visual impairment, overwhelming fatigue, or significant bowel or bladder impairment in an otherwise minimally impaired patient

Please select the best option that applies to [name12]'s mobility.

- ◯ Normal: functionally normal with no limitations on activity or lifestyle_ May have minor abnormality on examination _ Course is relapsing-remitting with a return to baseline with or without treatment_ Are not treated with any ongoing therapy for MS
- ◯ Mild disability: mild symptoms or signs_ Have definite findings such as sensory abnormalities, mild bladder impairment, minor incoordination, weakness, or fatigue_ No visible abnormality of walking_ The pattern of disease is relapsing-remitting, but may not have a full return to baseline following attacks_ May use ongoing therapy such as amantadine, baclofen, or oxybutynin
- ◯ Moderate disability: visibly abnormal walking but do not require walking aids _ The pattern of disease is relapsing-remitting or progressive
- ◯ Early cane: intermittent use of cane_ Other forms of unilateral support including splint, brace, or crutch _ Use unilateral support primarily for longer distances, but are able to walk at least 25 feet without it_ The pattern of disease is relapsing-remitting or progressive
- ◯ Late cane: dependent on a cane or other forms of unilateral support and cannot walk 25 feet without such support _ Patients may hang on to furniture inside their homes or touch the wall when walking in clinic_ May use a scooter for greater distances (e.g., malls) _ Pattern of disease is relapsing remitting or progressive
- ◯ Bilateral support: requires bilateral support to walk 25 feet_ May use two canes or two crutches or a walker _ May use a scooter for greater distances_ Pattern of disease is relapsing-remitting or progressive
- ◯ Confined to wheelchair: essentially confined to a wheelchair or scooter _ May be able to take a few steps but are unable to ambulate 25 feet, even with bilateral support _ May show further progression including worsening hand function or inability to transfer independently
- ◯ Unclassifiable: does not fit the above classifications_ Significant memory or visual impairment, overwhelming fatigue, or significant bowel or bladder impairment in an otherwise minimally impaired patient

Please select the best option that applies to [name13]'s mobility.

- ◯ Normal: functionally normal with no limitations on activity or lifestyle_ May have minor abnormality on examination _ Course is relapsing-remitting with a return to baseline with or without treatment_ Are not treated with any ongoing therapy for MS
- ◯ Mild disability: mild symptoms or signs_ Have definite findings such as sensory abnormalities, mild bladder impairment, minor incoordination, weakness, or fatigue_ No visible abnormality of walking_ The pattern of disease is relapsing-remitting, but may not have a full return to baseline following attacks_ May use ongoing therapy such as amantadine, baclofen, or oxybutynin
- ◯ Moderate disability: visibly abnormal walking but do not require walking aids _ The pattern of disease is relapsing-remitting or progressive
- ◯ Early cane: intermittent use of cane_ Other forms of unilateral support including splint, brace, or crutch _ Use unilateral support primarily for longer distances, but are able to walk at least 25 feet without it_ The pattern of disease is relapsing-remitting or progressive
- ◯ Late cane: dependent on a cane or other forms of unilateral support and cannot walk 25 feet without such support _ Patients may hang on to furniture inside their homes or touch the wall when walking in clinic_ May use a scooter for greater distances (e.g., malls) _ Pattern of disease is relapsing remitting or progressive
- ◯ Bilateral support: requires bilateral support to walk 25 feet_ May use two canes or two crutches or a walker _ May use a scooter for greater distances_ Pattern of disease is relapsing-remitting or progressive
- ◯ Confined to wheelchair: essentially confined to a wheelchair or scooter _ May be able to take a few steps but are unable to ambulate 25 feet, even with bilateral support _ May show further progression including worsening hand function or inability to transfer independently
- ◯ Unclassifiable: does not fit the above classifications_ Significant memory or visual impairment, overwhelming fatigue, or significant bowel or bladder impairment in an otherwise minimally impaired patient

Please select the best option that applies to [name14]'s mobility.

- ◯ Normal: functionally normal with no limitations on activity or lifestyle_ May have minor abnormality on examination _ Course is relapsing-remitting with a return to baseline with or without treatment_ Are not treated with any ongoing therapy for MS
- ◯ Mild disability: mild symptoms or signs_ Have definite findings such as sensory abnormalities, mild bladder impairment, minor incoordination, weakness, or fatigue_ No visible abnormality of walking_ The pattern of disease is relapsing-remitting, but may not have a full return to baseline following attacks_ May use ongoing therapy such as amantadine, baclofen, or oxybutynin
- ◯ Moderate disability: visibly abnormal walking but do not require walking aids _ The pattern of disease is relapsing-remitting or progressive
- ◯ Early cane: intermittent use of cane_ Other forms of unilateral support including splint, brace, or crutch _ Use unilateral support primarily for longer distances, but are able to walk at least 25 feet without it_ The pattern of disease is relapsing-remitting or progressive
- ◯ Late cane: dependent on a cane or other forms of unilateral support and cannot walk 25 feet without such support _ Patients may hang on to furniture inside their homes or touch the wall when walking in clinic_ May use a scooter for greater distances (e.g., malls) _ Pattern of disease is relapsing remitting or progressive
- ◯ Bilateral support: requires bilateral support to walk 25 feet_ May use two canes or two crutches or a walker _ May use a scooter for greater distances_ Pattern of disease is relapsing-remitting or progressive
- ◯ Confined to wheelchair: essentially confined to a wheelchair or scooter _ May be able to take a few steps but

are unable to ambulate 25 feet, even with bilateral support _ May show further progression including worsening hand function or inability to transfer independently

◯ Unclassifiable: does not fit the above classifications_ Significant memory or visual impairment, overwhelming fatigue, or significant bowel or bladder impairment in an otherwise minimally impaired patient

Please select the best option that applies to [name15]'s mobility.

◯ Normal: functionally normal with no limitations on activity or lifestyle_ May have minor abnormality on examination _ Course is relapsing-remitting with a return to baseline with or without treatment_ Are not treated with any ongoing therapy for MS

◯ Mild disability: mild symptoms or signs_ Have definite findings such as sensory abnormalities, mild bladder impairment, minor incoordination, weakness, or fatigue_ No visible abnormality of walking_ The pattern of disease is relapsing-remitting, but may not have a full return to baseline following attacks_ May use ongoing therapy such as amantadine, baclofen, or oxybutynin

◯ Moderate disability: visibly abnormal walking but do not require walking aids _ The pattern of disease is relapsing-remitting or progressive

◯ Early cane: intermittent use of cane_ Other forms of unilateral support including splint, brace, or crutch _ Use unilateral support primarily for longer distances, but are able to walk at least 25 feet without it_ The pattern of disease is relapsing-remitting or progressive

◯ Late cane: dependent on a cane or other forms of unilateral support and cannot walk 25 feet without such support _ Patients may hang on to furniture inside their homes or touch the wall when walking in clinic_ May use a scooter for greater distances (e.g., malls) _ Pattern of disease is relapsing remitting or progressive

◯ Bilateral support: requires bilateral support to walk 25 feet_ May use two canes or two crutches or a walker _ May use a scooter for greater distances_ Pattern of disease is relapsing-remitting or progressive

◯ Confined to wheelchair: essentially confined to a wheelchair or scooter _ May be able to take a few steps but are unable to ambulate 25 feet, even with bilateral support _ May show further progression including worsening hand function or inability to transfer independently

◯ Unclassifiable: does not fit the above classifications_ Significant memory or visual impairment, overwhelming fatigue, or significant bowel or bladder impairment in an otherwise minimally impaired patient

---

We understand you have a first-degree relative with MS. Please answer the following about your relationship with this person.

Compared to the others you mentioned, how close do you feel to this person?

◯ Especially close
◯ Not especially close

On average, how often do you speak with this person?

◯ Daily
◯ Weekly
◯ Monthly
◯ Less often
◯ Don't know

How far do you live from this person?

◯ Same house
◯ 1-5 miles
◯ 5-15 miles
◯ 16-50 miles
◯ 50+ miles

How old is each person? If you don't know the exact age, what is your best guess plus or minus 5 years?

[name1] ______________________

[name2] ______________________

[name3] ______________________

[name4] ______________________

[name5] __________________________________

[name6] __________________________________

[name7] __________________________________

[name8] __________________________________

[name9] __________________________________

[name10] __________________________________

[name11] __________________________________

[name12] __________________________________

[name13] __________________________________

[name14] __________________________________

[name15] __________________________________

Have you done any of the following to try and improve your health in the last 3 months?

If you do not smoke, do not take medications, or did not have any doctor's appointments in the last 3 months, please click "Not Applicable"

Cut back on smoking?
- ◯ Yes
- ◯ No
- ◯ Not applicable

Exercised at least 3 times per week?
- ◯ Yes
- ◯ No

Regularly taken your medications, meaning missed them less than once per month?
- ◯ Yes
- ◯ No
- ◯ Not applicable

Attended all doctor appointments?
- ◯ Yes
- ◯ No
- ◯ Not applicable

Have your contacts tried to improve their health in the last 3 months? Has anyone cut back smoking?If they do not smoke, please click "Not Applicable'.

	Yes	No	Don't know	Not applicable
[name1]	○	○	○	○
[name2]	○	○	○	○
[name3]	○	○	○	○
[name4]	○	○	○	○
[name5]	○	○	○	○
[name6]	○	○	○	○
[name7]	○	○	○	○
[name8]	○	○	○	○
[name9]	○	○	○	○
[name10]	○	○	○	○
[name11]	○	○	○	○
[name12]	○	○	○	○
[name13]	○	○	○	○
[name14]	○	○	○	○
[name15]	○	○	○	○

Do your social contacts exercise 3 times per week within the last 3 months?

	Yes	No	Don't know
[name1]	○	○	○
[name2]	○	○	○
[name3]	○	○	○
[name4]	○	○	○
[name5]	○	○	○
[name6]	○	○	○
[name7]	○	○	○
[name8]	○	○	○
[name9]	○	○	○
[name10]	○	○	○
[name11]	○	○	○
[name12]	○	○	○
[name13]	○	○	○
[name14]	○	○	○
[name15]	○	○	○

Do your contacts regularly take their medications, meaning missed them less than once per week in the last 3 months?If a person does not take medications, please select "Not applicable".

	Yes	No	Don't know	Not applicable
[name1]	◯	◯	◯	◯
[name2]	◯	◯	◯	◯
[name3]	◯	◯	◯	◯
[name4]	◯	◯	◯	◯
[name5]	◯	◯	◯	◯
[name6]	◯	◯	◯	◯
[name7]	◯	◯	◯	◯
[name8]	◯	◯	◯	◯
[name9]	◯	◯	◯	◯
[name10]	◯	◯	◯	◯
[name11]	◯	◯	◯	◯
[name12]	◯	◯	◯	◯
[name13]	◯	◯	◯	◯
[name14]	◯	◯	◯	◯
[name15]	◯	◯	◯	◯

Did your contacts attend all doctor's appointments in the last 3 months?If a person did not have doctor appointments, please select "Not applicable".

	Yes	No	Don't know	Not applicable
[name1]	◯	◯	◯	◯
[name2]	◯	◯	◯	◯
[name3]	◯	◯	◯	◯
[name4]	◯	◯	◯	◯
[name5]	◯	◯	◯	◯
[name6]	◯	◯	◯	◯
[name7]	◯	◯	◯	◯
[name8]	◯	◯	◯	◯
[name9]	◯	◯	◯	◯
[name10]	◯	◯	◯	◯
[name11]	◯	◯	◯	◯
[name12]	◯	◯	◯	◯
[name13]	◯	◯	◯	◯
[name14]	◯	◯	◯	◯
[name15]	◯	◯	◯	◯

How far does each person live from you?

For people less than 1 mile, click on 1-5 miles.

	Same house	1-5 miles	5-15 miles	16-50 miles	50+ miles
[name1]	◯	◯	◯	◯	◯
[name2]	◯	◯	◯	◯	◯
[name3]	◯	◯	◯	◯	◯
[name4]	◯	◯	◯	◯	◯
[name5]	◯	◯	◯	◯	◯
[name6]	◯	◯	◯	◯	◯
[name7]	◯	◯	◯	◯	◯
[name8]	◯	◯	◯	◯	◯
[name9]	◯	◯	◯	◯	◯
[name10]	◯	◯	◯	◯	◯
[name11]	◯	◯	◯	◯	◯
[name12]	◯	◯	◯	◯	◯
[name13]	◯	◯	◯	◯	◯
[name14]	◯	◯	◯	◯	◯
[name15]	◯	◯	◯	◯	◯

Supplementary Methods 2: Equations for Social Network Metrics

$$\text{Size} = N$$

where N is the number of nodes, not including the ego.

$$\text{Density} = \frac{2L}{N(N-1)}$$

where L is the number of ties, and N is the number of nodes, not including the ego.

$$\text{Constraint of i's network} = (p_{ij} + \sum_q p_{iq} \times p_{qj})^2$$

where i is the ego, q and j are alters, p_{ij} is the proportional strength of i's relation with j, p_{iq} is the proportional strength of i's relation with q, and p_{qj} is the proportional strength of q's relation with j.

$$\text{Effective size of } i'\text{s network } = \sum_j \left[1 - \sum_q p_{iq} \times m_{jq} \right], q \neq i, j$$

where i is the ego, q and j are alters, and $\sum_q p_{iq} \times m_{jq}$ measures the portion of i's relationship with j that is redundant to i's relationships with other primary contacts.

$$\text{Maximum Degree} = L_{\max}$$

where $L_{\max}$ is the highest number of ties incident on a single node, not including the ego.

$$\text{Mean Degree} = \frac{L_1 + L_2 + \cdots + L_N}{N}$$

where L is the number of ties from each node and N is the number of nodes, not including the ego.

Supplementary Figure 1: Map of Participants' Location

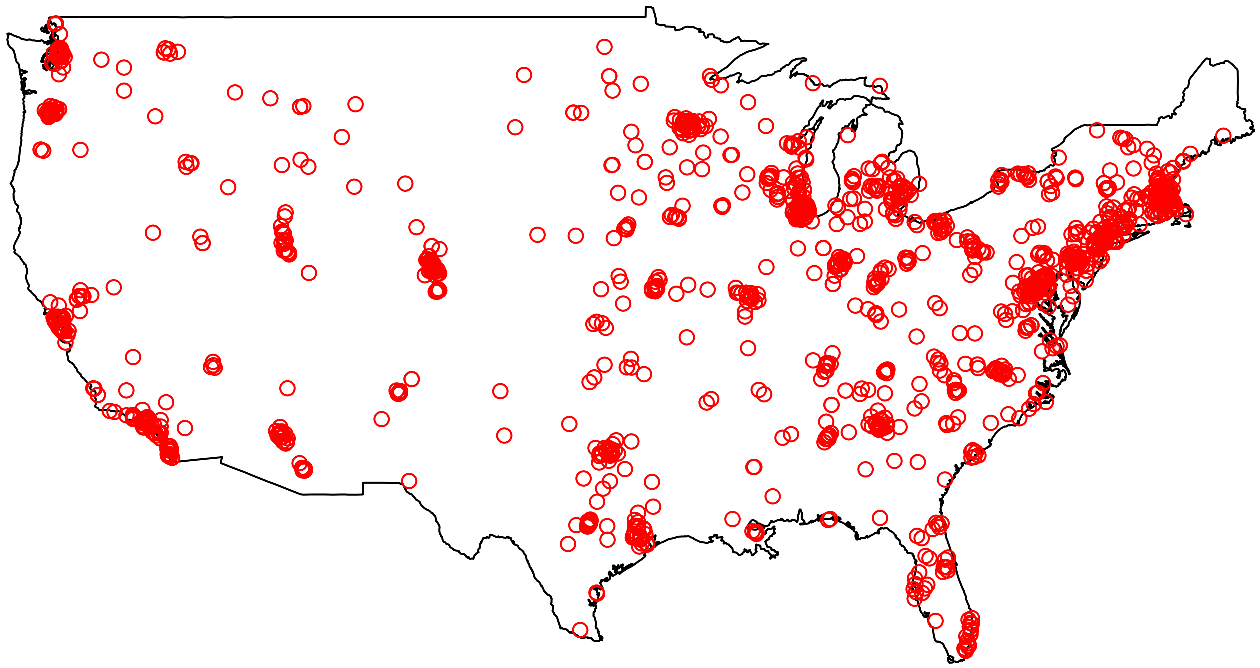

Social networks were examined in 1493 participants of the Genes and Environment in Multiple Sclerosis (GEMS) study across the United States.

Supplementary Table 1: Regression analysis of Genes and Environment Risk Score Components and Network Size

Variable	Beta	Standard Error	P-value [a]	n
**All Subjects**				
Genes and Environment Risk Score	0.815	0.192	2.43E-05	999
Environmental Risk Score	1.24	0.333	2.00E-04	999
Genetic Risk Score	0.561	0.229	0.0146	999
Mononucleosis	0.905	0.387	0.0197	1473
Male	-1.87	0.42	8.71E-06	1491
Smoking	-1.28	0.586	0.0291	1471
**Asymptomatic**				
Genes and Environment Risk Score	0.916	0.197	3.76E-06	920
Environmental Risk Score	1.34	0.34	9.44E-05	920
Genetic Risk Score	0.653	0.235	0.00552	920
Mononucleosis	1.13	0.4	0.00476	1364
Male	-1.91	0.431	9.91E-06	1376
Smoking	-1.53	0.601	0.0109	1363
**MS**				
Genes and Environment Risk Score	-0.291	0.856	0.734	79
Environmental Risk Score	0.227	1.42	0.873	79
Genetic Risk Score	-0.584	1.07	0.586	79
Mononucleosis	-1.64	1.51	0.277	109
Male	-1.23	1.8	0.495	115
Smoking	2.29	2.51	0.363	108
**Women**				
Genes and Environment Risk Score	0.8	0.239	8.67E-04	814
Environmental Risk Score	1.16	0.605	0.0547	814
Genetic Risk Score	0.717	0.259	0.00569	814
Mononucleosis	0.941	0.439	0.0324	1190
Male	-1.62	0.694	0.0194	1189
**Men**				
Genes and Environment Risk Score	-0.001	0.441	0.999	185
Environmental Risk Score	-0.036	1.06	0.973	185
Genetic Risk Score	0.006	0.471	0.99	185
Mononucleosis	0.328	0.77	0.671	283
Male	0.28	0.985	0.776	282

[a] Adjusted for age.
